# Supplementary material for: Causal Relationship between Aging and Anorexia Nervosa: A White-Matter-Microstructure-Mediated Mendelian Randomization Analysis
Source: Biomedicines. 2024 Aug 16;12(8):1874. doi: 10.3390/biomedicines12081874 (PMC11351342; doi:10.3390/biomedicines12081874)
Supplement: Supplementary file 1 [file biomedicines-12-01874-s001.zip › Supplementary Figure/Figure S4/Singe SNP plot of MR analysis for TL to ubm-b-1829.pdf]

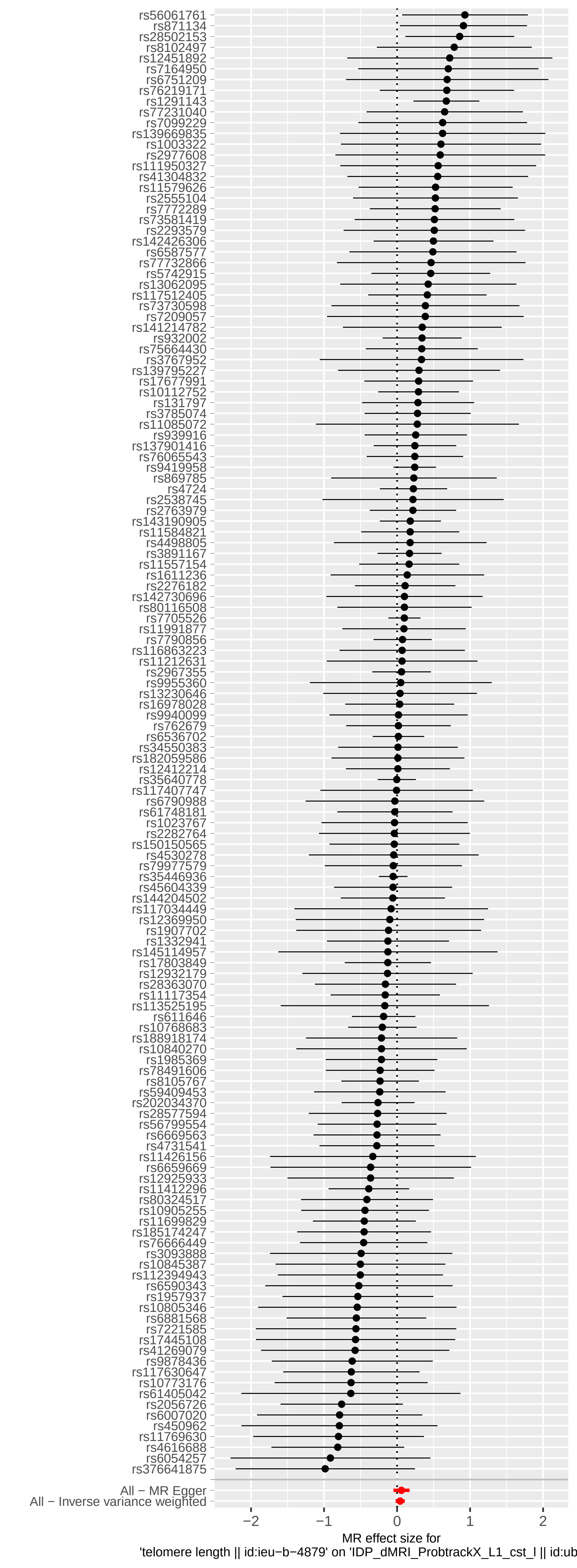

rs56061761
rs871134
rs28502153
rs8102497
rs12451892
rs7164950
rs6751209
rs76219171
rs1291143
rs77231040
rs7099229
rs139669835
rs1003322
rs2977608
rs111950327
rs41304832
rs11579626
rs2555104
rs7772289
rs73581419
rs2293579
rs142426306
rs6587577
rs77732866
rs5742915
rs13062095
rs117512405
rs73730598
rs7209057
rs141214782
rs932002
rs75664430
rs3767952
rs139795227
rs17677991
rs10112752
rs131797
rs3785074
rs11085072
rs939916
rs137901416
rs76065543
rs9419958
rs869785
rs4724
rs2538745
rs2763979
rs143190905
rs11584821
rs4498805
rs3891167
rs11557154
rs1611236
rs2276182
rs142730696
rs80116508
rs7705526
rs11991877
rs7790856
rs116863223
rs11212631
rs2967355
rs9955360
rs13230646
rs16978028
rs9940099
rs762679
rs6536702
rs34550383
rs182059586
rs12412214
rs35640778
rs117407747
rs6790988
rs61748181
rs1023767
rs2282764
rs150150565
rs4530278
rs79977579
rs35446936
rs45604339
rs144204502
rs117034449
rs12369950
rs1907702
rs1332941
rs145114957
rs17803849
rs12932179
rs28363070
rs11117354
rs113525195
rs611646
rs10768683
rs188918174
rs10840270
rs1985369
rs78491606
rs8105767
rs59409453
rs202034370
rs28577594
rs56799554
rs6669563
rs4731541
rs11426156
rs6659669
rs12925933
rs11412296
rs80324517
rs10905255
rs11699829
rs185174247
rs76666449
rs3093888
rs10845387
rs112394943
rs6590343
rs1957937
rs10805346
rs6881568
rs7221585
rs17445108
rs41269079
rs9878436
rs117630647
rs10773176
rs61405042
rs2056726
rs6007020
rs450962
rs11769630
rs4616688
rs6054257
rs376641875
All − MR Egger
All − Inverse variance weighted
−2
−1
0
1
2
MR effect size for
'telomere length || id:ieu−b−4879' on 'IDP_dMRI_ProbtrackX_L1_cst_l || id:ub
